# A Future Forecasting for FMCG Firm Performance in Covid-19: An Investigation of Consumer and Business Survival

**DOI:** 10.3389/fpubh.2021.723084

**Published:** 2021-09-17

**Authors:** Yunpeng Sun, Ying Li, Yujing Wang, Dayang Jiang, Xiaojun Liu

**Affiliations:** School of Economics, Tianjin University of Commerce, Tianjin, China

**Keywords:** Malaysian economy, GDP, Covid-19, companies efficiency, customer survival retention

## Abstract

The Covid-19 outbreak has spread over the world, limiting population and trade, causing job losses, and forcing businesses to close. The study's goal is to look at Covid-19's pandemic and consumer survival as a mediator for the future of running a business when FMCG companies are doing well. The researchers employed a basic random sampling strategy to do a layered transverse evaluation of samples. EFA, CFA, and SEM are used to choose data collection techniques for participants at Covid-19. According to the data, Malaysia has resulted in job losses, business growth, and customer satisfaction retention, as well as an increase in unemployment, company closures, and a drop in overall GDP. The impact of the Covid-19 epidemic on survival, production, and GDP has been demonstrated. Incorporating technology into all aspects of a company's working practices reveals the necessity and capacity of the organization to adapt to new scenarios.

## Introduction

The new Covid-19 sparked a global outbreak that began in Wuhan, China, and has since spread to 216 countries. As of mid-May 2020, there are 4,125,533 cases globally with 280,965 deaths (1). Investing in vaccine development, on the other side, has reduced global cases to 111,419,939 cases and 2,470,772 deaths as of late February 2021 (2). The Spanish flu is the most recent epidemic to interrupt business and social events. The pandemic ushered in a new way of life, both professionally and emotionally (3). The trade war between the United States and China, as well as the looming trade war, are the major sources of concern and dread in the business sector in 2019. The effects of the trade war on the global economy have alarmed business executives, and experts are divided on the subject (4). The International Monetary Fund (IMF) also weighed in on the subject, anticipating a global growth rate of 3.4% (5). Like no other, the Covid-19 outbreak struck damage (6). In a volatile market climate, essential decisions for survival are made, including as layoffs, salary cuts of up to 75%, and unpaid leave (7). Within a week following the pandemic's announcement, global financial markets plummeted, with stock markets losing about USD 6 trillion (8). While the unemployment rate in the United States reached its highest level of 14.7% since the Great Depression (9). Since crude oil prices have decreased, Malaysia is not immune to the issue. Despite Malaysia's estimate of $57, the price of crude oil is $54.77 in January 2021, and it is quite volatile. To compensate for the price shutdown had a substantial impact (10). Businesses have had to shut down operations as a result of the epidemic, resulting in the loss of a large number of jobs and lower productivity, notably among big makers of consumable products and services. The epidemic highlighted vulnerabilities in most nations' labor forces, notably in Malaysia, resulting in the obsoletement of numerous occupations and therefore unemployment. It also provided voice to technology-focused businesses, and certain technical tools became the new standard for hosting and organizing corporate meetings, distributing work, and assessing and stimulating staff performance. While the disease is wreaking havoc on people's health, it is also indirectly training people's brains, particularly those of firms and governments throughout the world, to embrace and fully incorporate a virtual manner of doing business and meeting people's wants into their systems (11, 12). The fast-moving-consumer-goods (FMCG) industry is one of Malaysia's most important industries, contributing significantly to the country's GDP, and is responsible for the manufacturing of essential products and services (13). In Malaysia, the food, beverage, and tobacco subsector of the FMCG business accounts for up to 5% of the country's total GDP in 2019. According to the Malaysian stock exchange, the FMCG business accounts for 17% of the equity in the country's market capitalization (14). This demonstrates the importance of the business to the Malaysian economy, as well as the study's focus on the pandemic's impact on the sector in relation to Covid-19's health risks. The global economic instability, the closure of businesses in Malaysia, and the impending recession all necessitated a study to investigate the influence of Covid-19 on company survival. Therefore, the research objective of this study mainly focuses on the FMCG market and Covid-19 outbreak. To the research question, this study aims to shed light on the effects of Covid-19 are how to affect the FMCG companies in Malaysia. To the novelty, this study uses EFA, CFA, and SEM to choose data collection techniques for participants at Covid-19.

Based on the former analysis, the contributions of this study are following: first, this study sheds lights on the Covid-19 are how to affect the FMCG companies in developing countries. Second, this study shown the effects of Covid-19 on Malaysian FMCG companies. To the implications, this study lays a solid foundation on the dangerous disease is how to affect the FMCG markets in developing countries. Moreover, this study may make future studies expand to other industries to provide a more comprehensive picture of the Covid-19's impact on the Malaysian economy and provide more insight into business operations.

## Literature Review

Covid-19 is linked to a slew of viruses that cause diseases including the common cold and Middle East Respiratory Syndrome (MERS-CoV). Despite efforts to stop the virus from spreading, global incidences of the Covid-19 virus continue to climb (15). Covid-19 is a worldwide epidemic that is impacting all sectors at the same time, and there is no hope until late 2020, when a vaccine will be released (16). However, in the first quarter of 2021, numerous significant advancements in the development of covid-19 vaccines are reported in nations such as the United States, Russia, and the United Kingdom, among others (2). Increased individualization, less religious meetings, and governments embracing new ways of engagement in economic, social, and political involvement are all possibilities for combating the spread (17).

### Covid-19 the International Financial System

The outbreak of the pandemic, China's economy is starting to catch up to that of the United States, with a gross domestic product (GDP) of $13.7 trillion. Regarding December 2019, China in the power cut of almost all procedure while involve (15). Due to the epidemic occurrence, the universal financial system is expected to contract by 2.4 % in 2020, with some analysts predicting even inferior (1.5%) in the first section of 2021. Moreover, Saudi Arabia's and Russia's oil price war had a detrimental influence on oil prices (4). 24.7 million jobs are lost as a result of the epidemic, with losses ranging from $860 billion to $3.4 trillion in revenue. A reduction in sales of this magnitude might trigger a global financial catastrophe and recession (18). Since the start of the Covid-19, the FTSE, Nikkei, and Dow Jones have all seen share prices plummet, indicating that the global stock market is rapidly declining due to global economic uncertainty (16). To set aside susceptible people, the US had to pump $2.2 trillion into the economy, and the UK did the same by paying up to 80% of workers' salaries to a trust fund. The biggest concern is that following the outbreak, economists anticipate an economic decline (9).

### Underpinning Theory, Profit Maximization Theory, and Survival-Base Theory

In his book The Prosperity of Nations, Adam Smith introduced profit maximization theory, stating that any firm will act in its own best interests in order to maximize profit from its economic activities. The survival-based hypothesis is initially offered by Herbert Spencer (19). The notion, which is popular in the twentieth century, stresses survival of the fittest, with every corporate organization utilizing every tactic available to achieve survival. According to the profit maximization concept, every business owner or firm would always act in their own best interests in order to maximize profit, secure long-term viability, and improve the total benefit to society (20). Businesses attempt to maximize profit by correlating marginal sales to marginal cost, according to the notion. According to the idea, as long as law and ethical custom are followed in the performance of the company's commercial activities, benefit maximization is the fundamental goal of the company (21). Survival-Founded Philosophy, on the other hand, is based on the survival of the fittest and emphasis that businesses must do whatever is legally feasible to prosper, compete, and thrive (22). It is normal for rivals to put out effort to establish the fittest organization that can adapt rapidly and effectively, according to survival-based philosophy. According to the notion, fierce market rivalry is advantageous to legitimate existence (23). The theory's applicability in corporate recovery is still relevant today, since failing businesses frequently encounter financial difficulties, labor attrition, failed items, market share losses, and other challenges. Job cutbacks/layoffs, wage cutbacks, the sale of an under-capacity asset, and product repositioning may all be necessary for an organization's resurrection (17). The fundamental aims of organizations are productivity, adaptability, and profitability, which assure their long-term viability (24). All actions performed by corporations, particularly in the aftermath of the Covid-19 epidemic, are crucial because they support these beliefs (profit maximization and survival).

### Hypotheses Development

The Covid-19 epidemic has had an influence on Malaysian businesses as well as the stock market. The Malaysian stock market has lost $5.9 billion in value, with further losses expected (6). Malaysian businesses have closed due to low consumption and supply issues (25). Now that client orders are at an all-time low, client retention is more challenging. Although the study (6) looked into Covid-19 and the economic crisis, it did not look at the influence on company survival, nor is it domesticated inside Malaysia's (FMCG) industry. In order to establish if Covid-19 has affected the chances of firm survival and consumer retention in the FMCG business, hypotheses one and two are developed.

**H**_**01**_**:** The pandemic of Covid-19 has an effect on the FMCG industry's ability to survive.

**H**_**02**_**:** The pandemic of Covid-19 has an effect on the FMCG sector's ability to survive due to consumer retention.

### Malaysian Firms' Performance During Covid-19

Over time, Malaysia FMCG industry has faced a number of challenges. The fall in consumer buying power in Malaysia as a result of the 2016 recession is an example. Covid-19 took a toll on the FMCG market, which is exacerbated by Dollar inaccessibility and poor macroeconomic conditions (26). One of the issues encountered by FMCG firms is the problem of volatility in all sectors of the business, causing many to lay off staff or impose forced leave without pay (25). The pandemic's influence on productivity is obvious in output volume in the FMCG industry, which has an indirect influence on market share. Some research has focused on Covid-19 and how it has effected those industries as well as the overall view of events in Malaysia (15, 27), but no research has yet to properly domesticate the study inside the FMCG industry, which is a gap that this study attempts to solve. Hypothesis three looks into the influence of Covid-19 on the FMCG industry's corporate survival and efficiency.

**H**_**03**_**:** The pandemic of Covid-19 has an impact on the FMCG sector's ability to survive because to a loss of production.

### FMCG Sector, Covid-19, Business Survival

Malaysia government and private sector work together to combat unemployment and create jobs for people of working age and unemployment will contribute to a rise in poverty, and the Covid-19 is already threatening to reverse the progress made in combating unemployment (28). The study by Adu (29) looked at the state of unemployment in a few industries in Malaysia, but it left out the FMCG market. This influenced the formulation of hypothesis four, which examined the possibility of increased unemployment in the FMCG sector in Malaysia as a result of the Covid-19.

**H**_**04**_**:** Covid-19 contribute negatively to unemployment/job loss and affect business survival in the FMCG sector.

### Technology Improvement During Covid-19

The adoptions of other modes of remote engagement under Covid-19 in order to maintain economic activity and prevent a complete shutdown are necessary to change firms' performance. Many businesses and government agencies are required to implement information technology (IT) as a result of Covid-19 (12). Technology adoption became a necessity, increasing operating costs. Is it a coincidence that the supply value of online video systems like Zoom, Microsoft Teams, Skype, and others has increased in tandem with Covid-19, or did it boost IT acceptance along the business value chain? Hypothesis 5 focuses on Covid-19, technology acceptability, and commercial survival.

**H**_**05**_**:** The Covid-19 epidemic exposed the level of corporate technology implementation and had an influence on company survival in the FMCG sector.

### During Covid-19, the Malaysian Financial System Is in a State of Flux

Malaysia practiced monetary depression in 2009 as a result of the global financial crisis, and again in 2016 as a result of a sharp drop in the international oil price. In 2019, the country is the world's 11th biggest producer of manganese, 11th biggest producer of tin, 12th biggest producer of bauxite, and 19th biggest producer of lime. The current Covid-19 outbreak is influencing the price of these precious metals, which are the primary source of foreign cash. Malaysia's economy is already suffering from the discrepancy in crude oil prices (6). Company closure leads in a reduction in taxes and earnings owing to the government, in addition to inadequate revenues to support the budget (10).

**H**_**06**_**:** Covid-19 has a detrimental impact on Malaysia's economy.

## Research Methods

The research is expressive in natural world because it assembles data by primary and secondary techniques to analysis hypothesis. To investigate the impact of the Covid-19 on the Malaysian economy, hypotheses one through five used survey responses, Hypothesis 6 is based on data from the Malaysian National Bureau of Statistics (www.dosm.gov.my) and the Central Bank of Malaysia (www.bis.org.com). The study population consists of 20 FMCG companies that are chosen based on their stock exchange ranking in Malaysia. Due of the Covid-19, which is restricting travel and generating social isolation, the questionnaire items are created in a Google format and delivered to the respondents. The collection of data related to these questionnaires took up to 4 months. The purpose of filling questionnaires from top level workers at each FMCG firm is to examine the Covid-19's long-term impact on Malaysian firms' performance. Customer satisfaction, company competitiveness, unemployment, and technology adoption-related questions are included in the questionnaires (30–33), which are important to assess the firms' performance in the short and long term.

### Collection of Instruments

The exploratory factor analysis (EFA) and confirmatory factor analysis (CFA) are used to assess homogeneity and data adequacy before testing hypotheses using the structural equation model (SEM).

To rationalize the application of SEM, researchers must examine the causal relationship between the study's measured, observed, and latent variables. Seventy-five to eighty-five percent of the data is returned throughout the 4-month data gathering period. The respondents' demographical situations have been represented in Table 1 and all the respondents belong to FMCG Firms' of Malaysia.

**Table 1:** The respondent's demographics.

		**Frequency**	**Legitimate %**	**Collective %**
Gender	Male	503	60	95.3
	Female	268	50	300
	Total	560	300	
Salary Range	Less than 5 million per annum	309	35.4	35.3
	5–30 million	383	56.9	63.5
	30–35 million	365	36.3	98.5
	35 million and above	30	3.2	300
	Total	660	300	
Highest qualification	BSc/HND	356	35.6	35.6
	MBA/MSc	300	55.8	69.5
	Postgraduate/Professional	342	30.6	390
	Certification			
	Total	670	250	
Department	Production/Supply chain	593	63.5	63.5
	Marketing and Sales	38	5.6	69.3
	Operations	350	30.87	300
	Total	560	200	

## Data Analysis and Results

### Assumptions for Multivariate Analysis

A SEM is used to assess the sample size, normality, missing values, and multi co-linearity hypotheses (34). Given that the sample size used for this analysis is 713, the recommended sample size of 200 (35) is reached 695. Questionnaire data is evaluated for skewness and kurtosis to check for normalcy within the (−1) and (+1) range (36). The data set had no outliers according to the frequency count and correlation analysis is used to check for multi-co-linearity. Moreover, The Harman's second approach, employs the CFA system to combine all 25 items used in the analysis into a single factor (Chi-square = 22.345, IFI = 0.62, CFI = 0.42, TLI = 0.63, NFI = 0.61, and RMSEA = 0.24) the result indicates a weak fit.

The traditional process bias rule is not broken in this research and EFA-based principal axis factoring eliminated redundant objects and investigated build loadings. The KMO and BTS tests are applied to evaluate homogeneity with (*p* = 0.05 and *p* = 0.000) suggested acceptance values (37). The KMO for the EFA of this study is 0.891, and BTS is 27988.297 (*p* = 0, and *p* = 0.05), to attain data adequacy as well as homogeneity. A one-dimensional analysis is used to test the model's fitness for SEM, and the measurements are assessed using composite reliability (CR), average variance extracted (AVE), Cronbach alpha, factor-loading, mean, and standard deviation. The Cronbach-Alpha reliability approach is used to determine the construct's dependability, and it must be more than 0.75, as Nunnally (38) recommends (see Table 2). Table 2 shows that the factor loadings for all 21 items are larger than 0.5, suggesting that the data loaded well and matched the measurement model well. The comparative fit index (CFI) measures how well the research model fits the null model, which assumes no relationships between model components. The CFI value for all constructs is more than 0.89, as shown in (Table 3), showing that the measurement model is well-fit (43). The CFI value shows that the model is fit. The CR approach is used to test each construct's internal consistency in terms of variance from an observable variable from its hidden component. Internal consistency is specified as a CR of <0.75; Table 2 reveals that all five components had greater values. The degree to which a concept captures variance from the overall amount of measurement error experience in a model is known as AVE. The threshold is chosen at 0.50 by Maravelakis (44) and non-violation is indicated in Table 2. As a consequence, homogeneity is obtained for the model utilized to test the study hypotheses using SEM. Just 25 things are deemed to be fit after being submitted to CFA as shown in Table 4 (CR-5, BS-2, BS-4, FP-3), and (COV-3, COV-5) are eliminated from customer retention, company survival, firm productivity, and the Covid-19, respectively.

**Table 2:** Determine the COVID-19 of evaluation points.

COV-1	Positive testing for the new covid-19 virus has been reported in my country.	
COV-2	This is the first time a pandemic of this size has struck my country.	Udofia et al. (39)
COV-3	My company's supply networks have been impacted by the virus.	
COV-4	The covid-19 virus has spread throughout the country.	
**FIRM TECHNOLOGY ADOPTION (FTA)**		
SD1	We have completely embraced technological adoption across the whole value chain of the organization.	
SD2	As a result of the epidemic, performance, and service delivery have become increasingly automated.	Türker (11)
SD3	I find it tough to use technology to deliver successfully.	
SD4	The company's overall performance is harmed as a result of the work-from-home policy.	
**BUSINESS SURVIVAL (BS)**		
OP1	Due of the epidemic, the corporation is facing a significant financial burden.	Bates (40)
OP2	As a result of the epidemic, there has been a major drop in the production of products and services.	Singh (41)
OP3	Poor manufacturing has resulted in low sales and returns during the last few months.	Korunka et al. (42)
OP4	To mitigate the impacts of the epidemic, we had to lay off employees.	Singh (41)
OP5	To cover salaries and/or wage payments, we had to restrict employee work hours every day.	Bates (40)

**Table 3:** Dimension CR, AVE, CFI models, and Chi-square test values.

**Measurement items**	**Constructs**	**CFI**	***R*** ^**2**^	**Mean**	**SD**	**Factor loading**	**Cronbach alpha**	**CR**	**AV E**
FTA1	Firm technology adoption		0.33	4.1	0.89	0.772^**^			
			1	8	1	^*^			
FTA2				4	0.879	0.786^**^			
			0.555	6	2	^*^			
FTA3				4	0.81	0.852^**^			
		0.871	0.441	0	8	^*^		0.71	0.65
FTA4		1		3.7	0.879	0.779^**^	0.763	1	6
			0.54	5	5	^*^			
FTA5				3.8	1.12	0.891^**^			
			0.507	4	1	^*^			
FTA6			2.38	3.9	1.17	0.895^**^			
			3	3	4	^*^			
COV1	Covid-19			4	1.03	0.773^**^			
			0.411	0	4	^*^			
COV2		0.89		3.7	1.17	0.846^**^		0.73	0.54
		4	0.8741	5	1	^*^	0.752	8	5
COV4				4	1.07	0.778^**^			
			0.655	4	4	^*^			
BS2	Business survival		0.44	4	1.06	0.804^**^			
		0.871	3	3	2	^*^		0.72	0.6
BS6		0	0.37	3.8	1.06	0.744^**^	0.833	8	3
			3	7	7	^*^			
U1	Unemployment			4	1.05	0.660^**^			
			0.359	5	3	^*^			
U2		0.871		3.9	1.06	0.710^**^	0.81	0.72	0.74
		5	0.775	9	8	^*^		1	5
U3			0.304	4.1	0.879	0.662^**^			
				2	2	^*^			
U4				4	0.81	0.754^**^			
			0.686	9	8	^*^			
U5				3.8	1.07	0.884^**^			
			0.557	4	1	^*^			
U6				3.9	1.07	0.774^**^			
			0.8749	6	4	^*^			
FP1	Firm productivity			4	1	0.562^**^			
			0.299	5	9	^*^			
FP2		0.871		3.7	1.06	0.614^**^		0.8	0.63
		2	0.419	7	8	^*^	0.833	3	2
FP4				3.6	1.09	0.784^**^			
			0.8792	4	7	^*^			
FP5				4	0.878	0.811^**^			
			0.301	5	3	^*^			
CR1	Customer retention			4.1	0.87	0.670^**^			
			0.432	3	5	^*^			
CR2				3.9	1.02	0.750^**^		0.71	0.65
		0.87	0.8796	7	1	^*^	0.81	0	0
CR3		5		4	0.873	0.692^**^			
			0.464	9	5	^*^			
CR4				4	1.19	0.650^**^			
			0.564	7	1	^*^			

*CR, composite reliability; AVE: average variance extracted; CFI, comparative fit indices; χ^2^, Chi-square Value*.

** *is 0.05 significance level;*

* *is 0.1 significance level*.

**Table 4:** The root mean square error of approximation and the CFA results.

	**_X2_**	**df**	**p**	**CFI**	**TLI**	**IFI**	**GFI**	**RMSEA**
Measurement model	3.121	290	0	0.894	909	0.89	0.879	0.08
Recommended value	≤ 2 or 3			>0.87	>0.87	>0.87	>0.87	<0.08

The comparative fit index (CFI) = 0.894, Tucker-Lewis index (TLI = 0.893), incremental fit index (IFI = 0.890), goodness of fit index (GFI = 0.879), chi-square (*X*^2^ = 3.121), degree of freedom (Df = 290), and root mean square error of approximation (RMSEA = 0.08), these factors in Table 5 indicate that the model is over the acceptable acceptance level, making it suitable for testing the hypothesis (45, 46).

**Table 5:** Model of the hypothesized testing.

**Hypothesized model**	***R*** ^**2**^	**Standardized effect**	***t*** **-value**	***P*** **-value**	**Remark**
	0.056	0.23(DE)	4.780	0	Effects that are both positive and direct
COV → FT → BS	0.327	0.453(IE)	12.322	0	Effects that are both positive and direct
COV → U → BS	0.304	0.74(IE)	15.244	0	Effects that are both positive and direct
COV → CR → BS	0.079	−0.53 (IE)	−7.245	0	Effects that are both positive and direct
COV → FT → BS	0.090	0.53(IE)	7.123	0	Effects that are both positive and direct

*DE, direct effect; IE, indirect effect; BS, business survival; FTA, firm technology; P = 0.05; where: DE, direct effect; IE, indirect effect; BS, business survival; FTA, firm technology; CR, customer retention; U, unemployment; COV, Covid-19*.

### Hypotheses Testing

**Figure 1:**
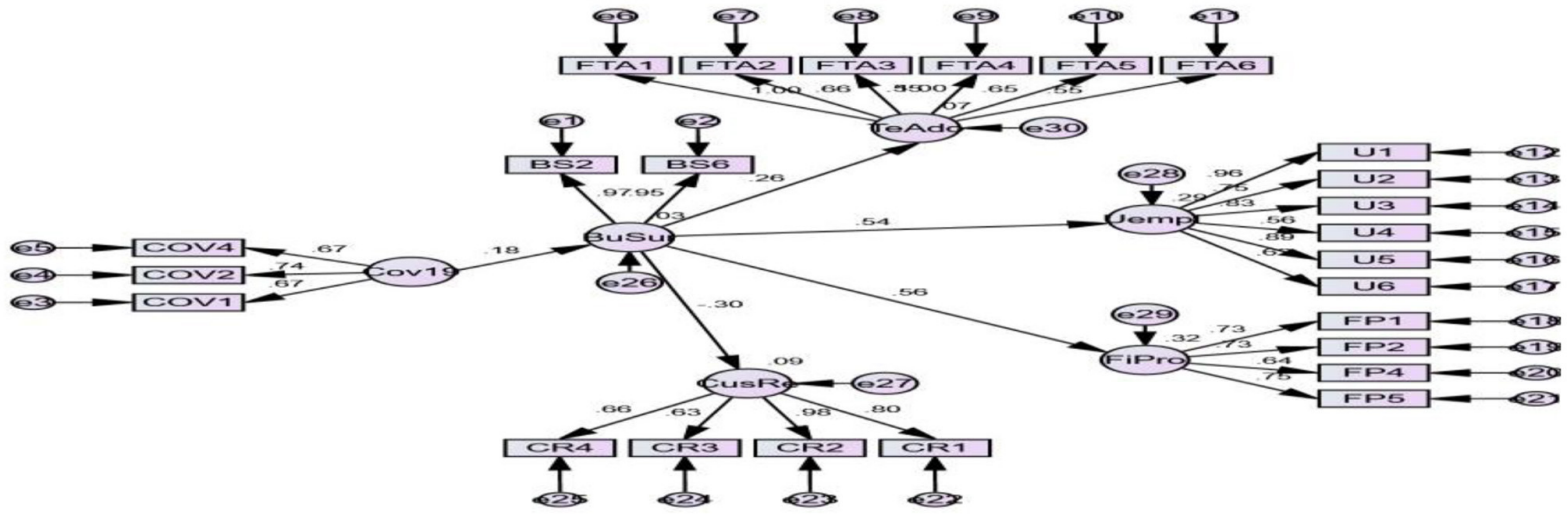
Standardized hypothesized structural equation model.

Only the first five hypotheses are estimated using SEM, as shown in Figure 1, which displays the direct, indirect, and standardized regression weights. In order to investigate the impact of the Covid-19 on Malaysia's economic prospects, the sixth hypothesis is tested using a narrative discourse that contrasted the third and fourth quarters of 2019 to the first and second quarters of 2020, based on secondary data from NBS and CBN. The hypothesis is accepted after the SEM analysis found that (H1) there is a clear relationship between the Covid-19 and FMCG company output (*t*-value = 3.679 at *p* = 0.005). This suggests that the unexpected outbreak of the Covid-19 would have a negative effect on the FMCG sector's business continuity and survival. The indirect hypotheses (H_2_-H_5_) look at how the Covid-19 affects the survival of firms in the FMCG market, using firm performance indicators including efficiency, unemployment, consumer retention, and technology adoption.

The results show that the Covid-19 has shook the activities of numerous FMCG firms, determining their success or failure. These assumptions are recognized since the analysis shows that the Covid-19 has a positive and indirect influence on company efficiency, unemployment, and company technology adoption (*t*-value = 12.234, *t*-value = 15.134, and *t*-value = 7.345 at *p* = 0.05). According to Hypothesis, the Covid-19 had a negative, indirect, and significant influence on FMCG customer retention and firm survival (*t*-value = −7.345 at *p* < 0.05). H02 is recognized as a result of the principal impacts. This might be due to the lockout, which makes it difficult for businesses and consumers to develop a steady transactional relationship, prompting customers to seek out alternative enterprises with significant web presences that can fill their demands fast during the lockdown.

**H**_**06**_**:** Covid-19 has a detrimental impact on Malaysia's economy

The Malaysian economy's prospects are determined by the GDP, inflation rate, and interest rate. The research utilized the GDP and inflation rate to compute the Malaysian economic forecast in order to estimate the influence of the Covid-19 on the Malaysian economy in comparison to the previous year 2019. This will demonstrate the economic impact of the pandemic in Malaysia and determine whether H06 will be accepted or denied.

Malaysia GDP increased by 1.87% in the first quarter of 2020, a decrease from the 2.55% rise in the fourth quarter of 2019. The drop in GDP in the first quarter of 2020 may be attributed to a halt in foreign trade. Similarly, GDP fell by −6.10% in the second quarter, compared to the previous quarters' rise and in comparison to the previous quarter, the third quarter saw a 3.62 % drop. However, the fourth quarter saw a 0.11 % increase in GDP, bringing the total GDP annual growth rate to 1.92% lower than the previous year. As a result, this drop demonstrates the negative impact Covid-19 has had on Malaysia economic outlook. Furthermore, from the last 5 months of 2019 to the last 5 months of 2020, the Malaysian inflation rate has been rising at an alarming rate. While the pattern of these increases has been constant since the beginning of 2019, it is impossible to say with certainty that the continuous rise in the year 2020 is due to the Covid-19. However, given the annual decrease in Malaysian GDP (−1.92) during the global pandemic in 2020, compared to the annual rise (2.27) in 2019, we can conclude that the Covid-19 has a negative effect on Malaysia economic outlook, leading to acceptance of the hypothesis.

## Conclusions and Recommendations

This study focuses on the viability of 2019 coronavirus disease companies and the success of FMCG companies. Six viewpoints are studied, five of which are analyzed by structural equation, and the sixth is analyzed by narrative discourse. All five hypotheses evaluated by SEM were correct. Due to the mandatory confinement of the Malaysian government, covid-19 has had an impact on the survival of enterprises in the FMCG industry. Companies forced to close down face liquidation and survival problems all over the world. In addition, 2019 coronavirus disease reveals the degree of technology use and survival of enterprises in the FMCG market. This is consistent with the survival-based mentality and profit maximization, because any company that cannot produce with maximum capacity or maximize profits is in danger of failure to survive and prosper. In the twenty-first century, today's market environment is developing at such an amazing speed. If technology is not fully integrated into business, it is difficult for enterprises to keep up. The data show that many FMCG enterprises have a low technology adoption rate in the whole value chain. According to the survey results, covid-19 has an impact on the unemployment rate and/or unemployment rate, which has an impact on the company survival of FMCG enterprises. This finding confirms these claims. In 2020 and 2019, coronavirus disease has an adverse impact on the unemployment rate in 19 countries, resulting in unemployment and loss of income. In FMCG 2019 coronavirus disease, covid-19 has an impact on enterprise productivity and survival. 2019 coronavirus disease the impact of 2019 coronavirus disease, consumer well-being and company survival rate have been negatively affected by cvid-19. Finally, the data show that covid-19 has a negative impact on Malaysia's annual GDP, and the economy is expected to decrease by 1.92% over the previous year. This is predictable because of the stagnation of international commerce and the blockade of global commerce. The country's GDP is declining and inflation is growing at an unprecedented rate, resulting in the loss of considerable value of the country's currency compared with international currencies such as the US dollar.

### Significances

Malaysian employees, company productivity, and unemployment rate were all affected by the Covid-19. Several nations, particularly industrialized ones, are, nevertheless, aiding firms in order to lessen the effects of the Covid-19. Further research can be done with a larger sample size on the same topic, and while this study focuses on the impact of the Covid-19 on the survival of FMCG companies, future studies may expand to other industries to provide a more comprehensive picture of the Covid-19's impact on the Malaysian economy and provide more insight into business operations. In order to compare the influence of the Covid-19 on firm survival and outcomes in established and emerging countries, more study is needed.

## Data Availability Statement

The original contributions presented in the study are included in the article/supplementary material, further inquiries can be directed to the corresponding author/s.

## Author Contributions

YS: conceptualization, methodology, data collection, writing, and data analysis. XL: supervision. DJ: original draft preparation. YW: visualization and investigation. YL: data collection and analysis. All authors contributed to the article and approved the submitted version.

## Conflict of Interest

The authors declare that the research was conducted in the absence of any commercial or financial relationships that could be construed as a potential conflict of interest.

## Publisher's Note

All claims expressed in this article are solely those of the authors and do not necessarily represent those of their affiliated organizations, or those of the publisher, the editors and the reviewers. Any product that may be evaluated in this article, or claim that may be made by its manufacturer, is not guaranteed or endorsed by the publisher.

## Abbreviations

EFA: exploratory factor analysis
CFA: confirmatory factor analysis
FTA: firm technology adoption
BS: business survival
SEM: structural equation model
KMO: Kaiser–Meyer–Olkin
BTS: Bartlett's-test of sphericity
CS: composite reliability
AVE: average variance extracted
CFI: comparative fit index
TLI: Tucker-Lewis index
RMSEA: root mean square error of approximation
IFI: incremental fit index
GFI: goodness of fit index
Df: degree of freedom.
